# Patient reported goals for medications for opioid use disorder: A theory of proximal goal attainment

**DOI:** 10.1016/j.rcsop.2023.100345

**Published:** 2023-10-06

**Authors:** Kenneth C. Hohmeier, Alina Cernasev, Christina Leibold, Todd M. Moore, Erica Schlesinger, Ileana Arce, Wesley Geminn, Marie Chisholm-Burns, Gerald Cochran

**Affiliations:** a University of Tennessee Health Science Center, College of Pharmacy, Department of Clinical Pharmacy and Translational Science, Nashville, TN 37211, USA; b University of Tennessee, Department of Psychology, Knoxville, TN 37996, USA; c Tennessee Department of Mental Health & Substance Abuse Services, Nashville, TN 37243, USA; d Oregon Health & Science University, Office of the Provost, Portland, OR 97239, USA; e University of Utah, Division of Epidemiology, Salt Lake City, UT 84112, USA

**Keywords:** Opioid use disorder, Patient reported outcomes, Patient-centered care, Medication for opioid use disorder

## Abstract

**Background:**

There exist substantial patient barriers to accessing medications for opioid use disorder (MOUD), including travel distance, stigma, and availability of MOUD providers. Yet, despite these barriers, there exists a subset of patients who possess the requisite motivation to seek and remain adherent to treatment.

**Objective:**

To explore patient-derived goals in MOUD treatment-adherent patients.

**Methods:**

This study used in-depth interviews with patients receiving methadone who were enrolled in opioid treatment programs (OTPs) across Tennessee. Participants were recruited from 12 different OTPs to participate in telephonic semi-structured interviews to a point of saturation. Participants had to be adherent to treatment, in treatment for 6 months or greater, and English speaking. Analysis occurred inductively using a constructivist approach to Grounded Theory.

**Results:**

In total, 17 patient interviews were conducted in the spring of 2021. Participants described goal setting across three general stages of treatment: (1) addressing acute physical and emotional needs upon treatment entry, (2) development of supportive structure and routine to develop healthy skills facilitated by treatment team, and (3) identifying and pursuing future-focused goals not directly linked to treatment. A Proximal Goals in MOUD Framework is introduced.

**Conclusion:**

In this qualitative study on patient reported goals in MOUD it was found that goals are transitory and relative to the stage of treatment. Further research is needed to better understand goal evolution over the course of treatment and its impact on treatment retention.

## 1 Background

Despite the availability of medication treatment for opioid use disorder (OUD) in the U.S.,^1^, ^2^, ^3^ patients with OUD continue to experience poor outcomes.4., 5, 6 This is in spite of the evidence that medications for OUD (MOUD) care serves to reduce opioid use, improve overall treatment retention, and reduce OUD mortality.^3^^,^^6^, ^7^, ^8^

There exist substantial patient barriers to accessing MOUD, including travel distance,^9^ stigma,^10^, ^11^, ^12^ and availability of MOUD providers.^13^, ^14^, ^15^, ^16^ In particular, methadone treatment is perhaps the most inaccessible form of MOUD given its limited distribution within opioid treatment programs, high degree of methadone-specific stigma, and access concerns, with a substantial segment of individuals driving over an hour to the methadone treatment facility most days of the week.^17^^,^^18^ Yet, despite these barriers to methadone, there exists a subset of patients who possess the requisite motivation to seek and remain adherent to methadone treatment.^19^, ^20^, ^21^, ^22^

Literature increasingly supports patient engagement in OUD treatment decisions to improve motivation, care retention, and medication adherence.^23^ Several models have been suggested to facilitate patient engagement, including shared decision making and using patient-reported goals (PRGs).^24^^,^^25^ PRGs may have distinct advantages over traditional, provider-defined treatment goals, as provider-defined goals may oversimplify the complexities of managing care for a patient with OUD and may value aspects of the care differently from their patients.^26^, ^27^, ^28^ However, complicating the issue is the fact that these patient goals likely shift as a patient moves from addiction through to the various stages of recovery.^17^ As Sanger et al. describe it, “…patients who no longer attend treatment programs and achieved sustainable recovery may have a different outlook on treatment goals compared to patients in the active phase of substance use disorder.”^29^

Despite its benefits, the practicality of such patient-centered care models for MOUD are lacking clear and actionable guidance, leaving clinicians without direction on how to effectively implement.^30^ There exists a need to explore how these goals relate to motivation to continue treatment, especially in those patients who are adherent to methadone despite barriers including accessibility, cost, and stigma – such as is the case for patients taking methadone for OUD.^19^, ^20^, ^21^, ^22^ And, a similar need to understand how patient reported treatment goals evolve over the time in the continuum of treatment. The objective of this study was to identify patient-reported goals and motivations for treatment retention in methadone treatment-adherent patients with OUD.

## 2 Material and methods

### 2.1 Overview

This study used in-depth interviews and a constructivist approach to Grounded Theory.^31^ All patients were adults (>18 years of age) who were actively receiving methadone for treatment of OUD at one of the 12 state-licensed opioid treatment programs in Tennessee. To participate, patients had to be adherent to treatment (proportion of days covered ≥80% and actively receiving counseling), enrolled in the opioid treatment program treatment for 6 months or greater, English speaking, and willing to share their stories.

### 2.2 Data collection and analysis

Patient interviews were conducted in the spring of 2021 by three researchers IA, AC, KC using a semi-structured interview guide that included questions related to patient experiences in clinic. IA was a female pharmacist post-graduate research assistant, AC is a qualitative researcher, and KH is college of pharmacy faculty member. The interview guide was developed by KC and AC, with input from opioid treatment program stakeholders including clinicians, counselors, and administrators. Participants were made aware of the opportunity to participate in the study by their on-site counselors. As an incentive for participation, the interview could be counted as one of their required counseling sessions. Study participants were also given a $50 gift card as compensation for their time. The project was approved by the Institutional Review Board.

Interviews were conducted over the telephone and recorded using a digital recording device until a point of saturation. Audio recordings were professionally transcribed by a third party to avoid any bias. Field notes were taken during the interviews and used along with transcribed interviews in data analysis.

Using a Grounded Theory approach, data were first analyzed with open coding. This was followed by axial coding to investigate relationships across emerging categories of codes. Finally, selective coding was used to integrate categories into a cohesive theory. NVivo 12® qualitative software was used to analyze transcripts line-by-line using open, axial, and selective coding. KH and CL refined the code book to and regularly met to review discrepancies and ensure reliability of the themes as they progressed.

## 3 Results

### 3.1 Demographics

In total 17 patient interviews were conducted. The average interview length was 65.6 min. Patient demographic data is presented in Table 1. Participants were from middle and east Tennessee, mostly white, and had a mean age of 42 years old.

**Table 1:** Demographics of Participating Patients: Gender, Age, Race, and Clinic Location.

**Race**	
White	13
African American	2
Declined to answer	2
Male	9
Female	8
**Age**	
20–29	3
30–39	3
40–49	9
50–59	1
60–69	1
**Location**	
Nashville area	12
Knoxville area	6

### 3.2 Proximal goals in MOUD framework overview

Participants described goal setting across three general stages of treatment: (1) addressing acute physical and emotional needs upon treatment entry, (2) development of supportive structure and routine to develop healthy skills facilitated by treatment team, and (3) identifying and pursuing future-focused goals not directly linked to treatment. However, not all participants articulated progression through all three stages.

**Fig. 1:**
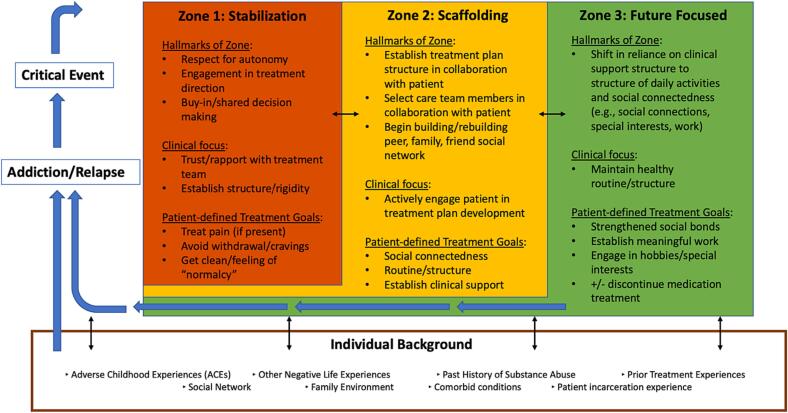
Proximal Goals in MOUD Framework.

The resulting Proximal Goals in MOUD Framework (Fig. 1) presents a visualization of how these stages, the individual's background, and the circumstances of their entry into treatment relate to one another.

### 3.3 Stages of proximal goals

Participants indicated that PRGs were linked to their success in treatment and emphasized the importance of being involved in the setting of treatment goals.

“*First of all, if you want to beat this, you've got to set yourself goals and you've got to stick to those goals, you've got to do it to make it work.*” (P14).

Participants noted that these goals were not fixed at a single point in time. Goals which were most proximal to the stage of recovery the patient was in were prioritized. Less proximal goals, like re-establishing severed relationships, were sometimes set as early on as treatment entry, but were not as readily achieved as more proximal goals like “achieving the right dose.”

*“My first goal was just to get stable and to be able to function and not have good days and bad days and stuff like that.”* (P5).

*“You set small [goals], and it'll lead up to bigger [goals]. …because I got two sons, and they're grown, and they got kids. I've got five grandkids. And I had to rebuild my relationship with my sons. And that took a while, and now we're in a good place.”* (P9).

Participants reported goals progressed in terms of difficulty and complexity over time.

*“… just getting up and making my bed every morning. Just something as simple as that. And I still do that. …It's hard to change the way you've been living your whole entire adolescent life and all of your adult life and just stop and stop talking to anybody and everybody you've ever met and trying to completely forget about that life and turn around and meet new people, better friends that are not using nor addicted to drugs, nor ever have been. It's hard to surround yourself with those kinds of people and stay on the right path.”* (P18).

Broadly, participants described goals across three main stages: “stabilization,” “scaffolding,” and “future focused” (Fig. 1).

#### 3.3.1 Stage 1: stabilization

The first stage was “stabilization,” which immediately followed the critical event which led the patient to treatment entry. It includes goals that were short-term and typically addressed acute concerns of the participant (e.g., withdrawal, cravings, pain). Social goals were lower priority, inwardly focused, and had concerns over autonomy, involvement in decisions, and overall rigidity of the program.

*“I don't really have no [long-term] goals for it. I mean, I don't wish on stars and stuff. I live one day at a time.”* (P1).

*“My timeline I had in my head literally when I started here, I'm like, I'm just going to go here like one year, maybe two, but then, when you get in there and you realize just to get a dose stabilized and get your right dosing amount, it takes like six to nine months, so that wasn't even realistic.”* (P3).

The patient-defined treatment goals for this stage include treatment of pain, avoiding withdrawal, and getting “clean”, and finding “normalcy”. Simple, and often short-term, goals were articulated.

“*The first week or so I noticed that my days would start differently. My day would just start better. I wasn't uncomfortable right away. I didn't wake up uncomfortable. I woke up in a better frame of mind. I woke up feeling better… And then, I noticed, as time went on, my sleep has even improved because I had sleep problems in the past, a lot of sleep problems. And it has got to the point now to where, I mean, I sleep eight hours a night… It made a lot of difference in my rest habits, a lot of difference in the way I- and I enjoy my meals more now. I'm more comfortable. So a lot of the normal things that a lot of people take for granted are better now for me.”* (P14).

The participants articulated the need to have a treatment team they could trust and establish a relationship early on in treatment.

“*You know, he's not just there to [say], ‘hey, did you go there, are you obeying the rules, are you- okay, goodbye.’ You know, it's, ‘How are you doing today? How are other things in your life? Do you have any other problems?’”* (P14).

“*Now, imagine that you've got to go to this person that you don't really feel comfortable with, and they're going to talk to you, and you're expected to talk to them about something that's the most sensitive and possibly damaging in many kinds of ways subject. And so, it won't work if you don't have a [care provider] that has a way of being understanding, non-judgmental, and always kind*.” (P13).

Some hallmarks of this stage include the respect for autonomy, engaging the patient in discussion of the direction of treatment, and subsequently creating buy-in using shared decision making.

“*My counselor and I have discussed my [treatment plan] quite frequently….”* (P13).

#### 3.3.2 Stage 2: scaffolding

The subsequent proximal stage, termed “scaffolding,” comprises goals that were centered on establishing new routines, treatment structure, a recovery care team, and re-establishing social connections. Participants described their transition from “stabilization” into “scaffolding” as desiring to co-establish their treatment plan in collaboration with care team members. Concepts of “structure,” “routine,” and “support” were used as participants described continuing their treatment and beginning to actively begin to rebuild positive peer, family, and friend networks.

*“I done suboxone. And, I would go take suboxone and take half my strip of suboxone and go trade it for meth and stuff, you know what I'm saying? Now, like, you know- [methadone] is controlled, you know what I'm saying? Like, because we got to go up there every day…”* (P12).

“*My quality of life changed, the people I surrounded myself with changed dramatically. The things I do on a day to day, my habits changed. My routine has definitely changed. I have much better, healthier routine now*.

“*And, well, I have a counselor, and if I got problems, I talk to them, and they kind of help me out. You know, to keep you on course. My life is a lot better when I've been going over there. You don't have no structure [at the beginning of treatment] and, you know, I got to have a kind of structure.”* (P9).

The “scaffolding” phase was distinct from the “stabilization” phase in that it seeks to form and reform the participant's life through engagement in routine, social connections, and healthy habits, whereas, “stabilization” focused primarily on avoidance of withdrawals, pain, and negative self-perceptions.

“*Like there had been times when I had tried to come off pills myself, and bought a bunch of methadone and tried to squirrel it away and slowly wean myself down. Now, it didn't work because I didn't have all the rest of the picture. Like the going to the clinic, the correct dose, the daily routine of being able to take it without worry of running out suddenly… There was too much stress trying to dose myself… You know, it didn't work.”* (P16).

Here participants desired a clinical focus of actively engaging with them in treatment plan development, including how often the patient needs to come into clinic, how many methadone patients may “take home,” and the structure and scheduling of counseling sessions. Here too, participants made mention of finding the “right” counselor in co-developing their structure, routines, and support.

“*I have a treatment plan, and [the clinician] always like brings me back if I start to get off track or off-center or less focus, she always brings me back to my treatment plan, reminds me of my goals, reminds me of things I can do. Like we worked on things like through my goals and my paperwork and even the classes I took there, they've helped me like realize like triggers and realize like how to manage those and realize like coping skills. So, I mean, there's several, you know, ways that she's like helped kind of guide me*.” (P3).

#### 3.3.3 Stage 3: future focused

Finally, participants described a stage beyond “scaffolding” which described goals of “future self”, including long-term aspirations such as licit work or travel. “Future self,” as distinct from “stabilization,” included concepts that were beyond a return to “normalcy,” which was defined as what life looked like to the participant prior to their addiction. In some cases, it also involved not defining oneself by addiction – and rather by other roles related to family, work, or hobby. As opposed to Stage 1 and 2 goals that were mostly or entirely rooted in clinical support, patient reported goals in this stage are rooted in the patient's newly developed routines and structures – which are more individualized to their own current needs and future desires. This includes strengthening social bonds, establishing meaningful work, engaging in hobbies and other special interests.

“*My life that I have now, the quality of life I have now, my wife that's wanting to start a family, I know to keep everything on track, I need to get up, come in, and stay on this routine and work the steps.”* (P6, Q8 & 9).

“*… I wanted my life to be manageable and I wanted to get like a part-time job, which I did end up doing. And I wanted to lose weight, which I'm working on that too now. You know, when you're in active addiction, you don't even think about like exercise or how to like improve yourself, and so that's nice that I can actually worry about going to the gym, or am I going to walk around the neighborhood with my daughter, you know, three times this week. So, [now] my goals are really just like all the different ways of being healthy and staying healthy pretty much*.” (P3).

Although social bonds may have been a goal in previous stages, it is in the “future self” stage that participants described deeper and strengthened relationships, sometimes beyond what they had been prior to addiction.

“*For the longest time, I didn't tell my kids like what was going on or what I had been through, but then I realized like they might benefit from my mistakes. And so I didn't hide anything. I've always been real honest and upfront and talked to them about it... And then also I've got friends, a close friend that is also supportive. So pretty much everybody that's around me knows my situation and is supportive of it*.” (P3).

Work and career aspirations was also a theme in this stage.

“Well, I think more than just trying to go after anything big, you just go after small things… and work towards them, like trying to finish my masters, so I can get that. So I'm probably six [credit] hours of away from that. I've been working on that. And then I'm also working on stuff for work to get other licenses so I can do more stuff.” (P5).

Identification of purpose or meaning was either implicitly or explicitly central to this stage. Such purpose sometimes related to building or maintaining positive family, other times it was what they did as “meaningful” work or as a hobby.

“*My father is sick with leukemia, so I've been staying up with him, trying to help take care of him, and it was just a job near him, and it kind of feels like it was meant to be because it's just worked out well. First job I've ever- or job I've had in a long time that I like getting up and going to… In my old job, I really just got to sit in the cubicle and never really got to do anything but sit there. Now I get to get out and meet people, and there's just something different all the time*.” (Respondent 5).

“*Once I complete the program I just want to be like an advocate and just to help African American people, so they know that there's actually help out there*.” (P4).

## 4 Discussion

This study explored patient reported goals of methadone in treatment-adherent patients at Opioid Treatment Programs in Tennessee. Participants described how that their reason for remaining adherent to MOUD was different at distinct stages throughout their treatment. Although the goals reported in this study paralleled patient goals described previously in the peer reviewed literature,^26^ the study adds to previous findings the concept that PRGs evolve over time as patient progresses through treatment – and that these goals are not only unique to the individual but to that individual's stage in treatment.

There has been an increasing interest in PRGs in OUD treatment over the past with a substantial overlap between study findings. Chai et al. reported six PRGs in MOUD treatment for youths aged 16–25 in Canada, including: (1) taper/stop MOUD, (2) avoid recreational substance use, (3) manage OUD symptoms/withdrawal, (4) live normal life, (5) improve mental health, and (6) gain employment.^25^ Similarly, Sanger et al. identified paralleling themes such as: (1) stop MOUD, (2) avoid illicit drugs, (3) live a “normal” life, (4) manage pain, (5) avoid OUD symptoms, (6) taper off MOUD, and (7) no changes in treatment.^29^

However, there exists divergence among some studies in identifying PRGs as well. For example, O'Reilly et al., identified only three goals, all of which were treatment-specific, including (1) continue, (2) stop immediately, (3) stop eventually.^32^ Marchand et al., 2020's results expanded on this slightly, including (1) reduced illegal activities, (2) stopping illicit opioid use, (3) increasing stability and routine, and (4) improved health.^33^

A recent systematic review compiled and categorized patient goals in OUD treatment across 12 domains and 43 goals.^27^ Although comprehensive in nature, the systemic review does not answer why there is divergence of results across these various studies or how these 44 goals factor over the course of treatment, nor did it find a unifying thread which holds together these goals. We propose that one potential unifying link is how these individual patient goals are both unique to the patient and their stage of treatment. This finding may be paramount to understanding their utility in clinical practice.

For example, the impact of patient goals on treatment success was investigated by Rosic et al. in a population of over 2000 participants who were receiving methadone or buprenorphine-naloxone treatment for OUD.^34^ Negative urine drug screen was the marker for treatment success. Six goals were reported by participants, including: (1) control cravings/withdrawal, (2) maintain medication dose, (3) live a “normal” life, (4) manage pain, (5) stay “clean”, (6) stop treatment. Surprisingly, the study found that none of the goals were associated with treatment success. Moreover, those participants who reported goals of staying “clean” or controlling cravings were less likely to be abstinent from opioids over the next 90 days than those who did not report those goals.

Understanding the larger continuum of how PRGs evolve over the course of a patient's treatment can help to reconcile such disparate goals, and may also explain why PRGs have yet to be associated with treatment retention. Similar to Rosic et al.,^34^ our study uncovered goals describing managing pain, living a “normal” life, staying “clean”, and managing pain. However, when these goals are mapped to the Proximal Goals in MOUD Framework one can see that each of the goals only capture “Stage 1: Stabilization.” Rather, it may be more appropriate to ask instead if participants had set goals in line with their current stage of treatment.

Importantly, the three stages of MOUD treatment in the Proximal Goals Framework terminate in a stage whereby the individual is centered on a routine involving social connectedness (e.g., family, friends), work (e.g., job, career), and special interests (e.g., volunteering, hobbies). This too mirrors findings from other work investigating addiction treatment retention generally. Both “social factors” and “purpose” have previously found to be in relationship with treatment retention.^35^ And, these items are also highlighted by SAMHSA as principles for recovery. Setting such goals too early in treatment may serve to be demotivating, as was alluded to by participants in this study. Monitoring progress toward these goals may assist providers, and there is precedent for the use of this information as part of a “clinical dashboard”.^36^

Although the concept of “proximal goals” has not been previously described in context of treatment for OUD, it borrows from established theory in educational psychology proposed by Lev Vygotsky.^37^ Vygotsky proposes that an individual can increase knowledge and skill across a continuum through a stepwise progression of increasing difficulty. The individual can most efficiently and effectively progress to the next “zone” (i.e., “stage”) through the assistance of a facilitator^37^ – in the present context, the clinical team. We propose that patients entering MOUD care may be largely incapable themselves of achieving goals in higher stages due to the goal's relative difficulty. However, with the assistance of a clinician trained in OUD treatment, patient goals may be achieved in a stepwise fashion as the patient progresses through recovery. It is this assignment of level of “difficulty” across these stages that the present study suggests, rather than a new set of patient-defined goals. Although navigation of these goals can be facilitated by an MOUD prescriber, it is worth noting the participants in this study greatly valued the support of “counselors” throughout their treatment.

Similar to the Transtheoretical Model of Change^38^ and the Model of Action Phases theories,^39^ the Proximal Goals in MOUD Framework also has a “staged” approach to understanding an individual's progression toward an end. However, whereas these theories center on an individual's progression toward adopting a particular behavior (i.e., seeking treatment for OUD), the present framework assumes an individual has already selected and is actively receiving said care (i.e., “action” and “maintenance”). In this way, the Proximal Goals in MOUD Framework guides clinicians in maintaining progress in treatment adherence and retention, rather than in pursuing treatment in the first place. Our novel framework also borrows from Goal Setting Theory,^43^^,^^44^ which underscores both the importance of setting “specific” goals and at the most appropriate “difficulty.” Goals which are too easy do not motivate, and goals which are too difficult demotivate.^43^^–45^Similarly, in our study, participants noted this phenomenon of not only choosing a specific goal but matching that goal to their stage of MOUD treatment.

Finally, this study emphasizes the importance of social connectedness and involvement throughout treatment, and especially in the latter stages of proximal goals. However, this remains a challenge for patients undergoing OUD treatment.^43^^,^^44^ Our findings underscore the critical nature of these familial and social relationships as both a goal and a facilitator for retention. And this has also been well-established in the peer reviewed literature.^43^^,^^44^

### 4.1 Limitations

This study has several limitations. This was a qualitative study and the sample only included participants from Tennessee, limiting generalizability of results. Convenience sampling was used to recruit patients, introducing potential bias via its inability to ensure collection of all views of the larger community. For instance, those patients who are willing to be interviewed about their MOUD experience may have a different view on goal setting than those unwilling. However, despite this limitation the resulting patient reported goals overlapped to a great extent those found in other populations across the Americas and Western Europe within the published literature. The development of a theoretical framework from these findings lends the results of this study to further testing and validation through cross-sectional and prospective experimental study designs.

## 5 Conclusion

In this qualitative study on patient reported goals in MOUD it was found that goals are transitory and relative to the stage of treatment. Further research is needed to better understand goal evolution over the course of treatment and its impact on treatment retention.

## Declaration of Competing Interest

The authors declare that they have no known competing financial interests or personal relationships that could have appeared to influence the work reported in this paper.
